# Ultra-short PROMs: clever or not?

**DOI:** 10.1038/sj.bjc.6605951

**Published:** 2010-11-09

**Authors:** C Zimmermann

**Affiliations:** 1 Palliative Care Program, Department of Psychosocial Oncology and Palliative Care, Princess Margaret Hospital, University Health Network, Toronto, Canada; 2 Campbell Family Cancer Research Institute, Ontario Cancer Institute, Princess Margaret Hospital, University Health Network, Toronto, Canada; 3 Division of Medical Oncology and Haematology, Department of Medicine, University of Toronto, Toronto, Canada

Patient-reported outcome measures (PROMs), defined as ‘a measurement of any aspect of a patient's health status that comes directly from the patient’ (Sloan *et al*, 2007), are increasingly being adopted into oncology research and clinical practice. In research, traditional outcomes, such as tumour response and mortality, are being supplemented with subjective evaluations of quality of life, satisfaction with care, psychological distress and symptom control. In clinical care, routine screening using PROMs is useful to identify clinical issues, focus discussions, identify problems, and track issues over time. Because of the perceived advantages in efficiency and feasibility, there is growing interest in short (5–20 item) or ultra-short (1–4 item) measures (Vodermaier *et al*, 2009). But how short is too short? Is there a point at which these measures become too brief to be meaningful?

In this issue of the *British Journal of Cancer*, Phillips *et al* (2010) report on a three-item patient-reported clinic satisfaction measure for young adults. Consecutive adult outpatients who were long-term survivors of childhood cancer completed a 16-item satisfaction with communication questionnaire; the data were used to develop a 3-item questionnaire, which was subsequently validated against the 16-item measure using data from a second study. The brief measure was highly correlated with the original longer measure in both the derivation and validation data sets, and had a fair capacity to discriminate between satisfaction and dissatisfaction using a specified cutoff point.

As Phillips *et al* point out, short questionnaires have obvious advantages. They take less time to complete, are less burdensome for patients, and have higher rates of completion and return. Missing data are a common problem in trials using PROMs and can lead to loss of power and biased results (Huntington and Dueck, 2005). Although there are various strategies to handle missing data, the best solution is prevention, including minimising patient burden with brief measures. In clinical practice, short PROMs interfere less with the efficient running of the clinic, particularly if they are to be completed before the appointment (such as for screening measures). In addition, they are less costly, because fewer personnel are necessary for administration, data entry and scoring. The advantages of short measures are perhaps best illustrated by pragmatic studies of acceptability. In a UK study assessing acceptability of common distress screening methods, only 1% of cancer clinicians indicated that they would be prepared to use a measure that was longer than 10 items (Mitchell *et al*, 2008).

Despite these advantages, there are also drawbacks to shorter questionnaires. The main doubt, particularly with ultra-short measures, is concerning their reliability and validity. In oncology, this issue has been investigated most thoroughly regarding measures of psychological distress, particularly depression. Mitchell (2007) reviewed 38 studies examining ultra-short PROMs for distress, and found that these tools were very good at excluding possible cases of depression (sensitivity 78%, negative predictive value 93%), but were poor at ruling in a suspected diagnosis (specificity 67%, positive predictive value 34%). This raises questions about what to do with the large numbers of patients who have a positive screen. Options include referring these patients directly to a professional who can diagnose depression, or administering a longer, more specific questionnaire; both of these options are again costly and/or burdensome, albeit for a smaller number of patients. In research situations, the lack of specificity of ultra-short distress measures is of even greater concern, and they are therefore not recommended for use in trials or other research studies (Vodermaier *et al*, 2009).

Outcomes of PROMs can range from individual physical symptoms to complex end points, such as quality of life. Ultra-short measures are likely most useful *in situations*, in which a clear single question is asked regarding a discrete outcome in a single domain. The numeric rating scale for pain is one example, in which a single-item scale has demonstrated validity and reliability, and is suitable for use in research settings (Breivik *et al*, 2008). Quality of life has physical, psychological, social and functional domains; although ultra-short and single-item measures have been validated, they are less reliable (stable) than multi-domain questionnaires, and their ambiguity limits their value as screening aids (Sloan *et al*, 2002). The more vague a single question or series of questions, the less likely it is that all patients will interpret the question in the same way, and the less likely it is that this will change following an intervention, because many aspects other than the intervention may have an influence. For example, in a study measuring the efficacy of a palliative care clinic on symptom control, there was significant improvement in 10 individual symptoms after the intervention, but a single-item measure of well-being did not change (Follwell *et al*, 2009).

The validation of ultra-short measures for depression has the advantage of an established ‘gold standard’ against which the measure can be compared. This is not the case for quality of life and especially not satisfaction with care, which will vary according to the aspect of satisfaction that is being measured. The measure developed by Phillips *et al* measures satisfaction with communication, and was validated by comparing the 3-item measure to the larger 16-item measure within which it was situated. As the authors acknowledge, the three questions may be answered differently if administered independently; comparison of the three-item measure against other measures of satisfaction and related constructs is needed for further validation.

Ultra-short measures may be developed *de novo*, or by eliminating all but a few items from a longer questionnaire. The roots of the 3-item measure developed by Phillips *et al* can be traced to a 55-item patient satisfaction questionnaire developed by Ware *et al* in the 1980s (Ware *et al*, 1983). This measure was shortened to a 28-item measure containing items specifically related to communication, which was then pilot tested and reduced to the 17-item patient satisfaction with communication questionnaire (Shilling *et al*, 2003), which was further reduced to 16 items (Absolom *et al*, 2006) and finally to 3. At each step of item reduction, information is lost, unless items are redundant, and it needs to be kept in mind that the questionnaire has the capacity to assess only the questions that are being asked. For example, the brief PROM developed by Phillips *et al* assesses understanding by asking whether medical terms were used that were not understood; however, this question may pertain more to physicians than to nurses or other staff, and a clinician may be difficult to understand without using medical terminology.

Ultimately, the choice of using an ultra-short measure or a longer one needs to take into account the advantages or disadvantages in the particular setting and circumstance that the measure will be used. Ultra-short measures are appropriate as research outcomes, particularly as secondary end points, as long as a discrete single domain is being measured. They also have a place as clinical screening measures, in which they have an advantage over longer measures in terms of feasibility and cost. However, there needs to be a realistic interpretation of the results, and a preestablished, concrete plan of what will be done with them. Little good comes from identifying a patient who may have depression or who may be dissatisfied with care, if there is no follow-up on an individual or an institutional basis.
